# Human TRIM5α mediated restriction of different HIV-1 subtypes and Lv2 sensitive and insensitive HIV-2 variants

**DOI:** 10.1186/1742-4690-3-79

**Published:** 2006-11-06

**Authors:** Patrick Kaumanns, Isabel Hagmann, Matthias T Dittmar

**Affiliations:** 1 Department of Virology, University of Heidelberg, Im Neuenheimer Feld 324, D-69120 Heidelberg, Germany

## Abstract

In order to characterize the antiviral activity of human TRIM5α in more detail human derived indicator cell lines over expressing wild type human TRIM5α were generated and challenged with HIV-1 and HIV-2 viruses pseudotyped with HIV envelope proteins in comparison to VSV-G pseudotyped particles. HIV envelope protein pseudotyped particles (HIV-1[NL4.3], HIV-1[BaL]) showed a similar restriction to infection (12 fold inhibition) compared to VSV-G pseudotyped viruses after challenging TZM-huTRIM5α cells. For HIV-2 a stronger restriction to infection was observed when the homologous envelope protein Env42S was pseudotyped onto these particles compared to VSV-G pseudotyped HIV-2 particles (8.6 fold inhibition versus 3.4 fold inhibition). It has been shown that HIV-2 is restricted by the restriction factor Lv2, acting on capsid like TRIM5α. A mutation of amino acid 73 (I73V) of HIV-2 capsid renders this virus Lv2-insensitive. Lv2-insensitive VSV-G pseudotyped HIV-2/I73V particles showed a similar restriction to infection as did HIV-2[VSV-G] particles (4 fold inhibition). HIV-2 envelope protein (Env42S)-pseudotyped HIV-2/I73V particles revealed a 9.3 fold increase in infection in TZM cells but remained restricted in TZM-huTRIM5α cells (80.6 fold inhibition) clearly indicating that at least two restriction factors, TRIM5α and Lv2, act on incoming HIV-2 particles. Further challenge experiments using primary isolates from different HIV-1 subtypes and from HIV-1 group O showed that wild type human TRIM5α restricted infection independent of coreceptor use of the infecting particle but to variable degrees (between 1.2 and 19.6 fold restriction).

## Findings

TRIM5 proteins of different species inhibit infectivity of a range of different retroviruses in a species-specific fashion [1,2]. Whereas rhesus macaque TRIM5α (rhTRIM5α) efficiently restricts human immunodeficiency virus type 1 (HIV-1) replication (up to 100 fold reduction in viral titer), the human homologue shows limited but reproducible activity against HIV-1 (2 to 3 fold reduction in viral titer), but restricts N-tropic strains of the murine leukemia virus (N-MLV) very efficiently [3-8]. Different human cell lines (e.g. HeLa, 293T, C134 cells) over expressing a HA-tagged human TRIM5α have been used to determine the efficiency of HIV-1 specific restriction. Ylinen and colleagues showed that HIV-2 particles are weakly restricted by human TRIM5α expressed in TE671 cells and efficiently restricted by rhesus TRIM5α [9], thus showing a similar phenotype as HIV-1 particles.

In addition to TRIM5α it was shown that a yet unidentified restriction factor expressed in human cells restricts early post entry steps of HIV-2 [10]. This factor, called Lv2, acts on incoming HIV-2 particles like TRIM5α but can be bypassed if VSV-G pseudotyped HIV-2 particles are used to challenge target cells [10-12].

The viral capsid of HIV-1 is the main target for the antiviral effect, since certain mutations in the capsid protein (for example exchange of glycine to valine or alanine at position 89, G89V and G89A respectively) have been shown to confer resistance to TRIM5α mediated restriction [5,13-15]. For HIV-2 it has been shown that particles encoding the amino acid valine at position 73 are insensitive to Lv2-mediated restriction [11].

Most published studies to detect post entry restrictions have used viral particles pseudotyped with vesicular stomatitis virus glycoprotein (VSV-G). This allows the determination of species-specific restrictions independent from the expression of the appropriate receptors for infection [16-19] and indicates an independence from the route of viral entry (plasma membrane fusion vs endocytotic uptake) for the observed restriction of HIV-1, whereas Lv-2 mediated restriction of HIV-2 is entry route dependent [10-12].

In order to use authentic viral particles (primary isolates from different subtypes, including HIV-1 group O) for the characterization of human TRIM5α mediated restriction, the indicator cell line TZM-bl [20] was stably transduced with a retroviral vector (LNCX2, Clonetech, Germany) encoding wild-type, non-tagged human TRIM5α (obtained from PD Bieniasz, [21]) and G418 resistant cells were selected. TZM-bl cells are HeLa-cell derivatives that express high levels of CD4 and both co-receptors CXCR4 and CCR5, and are stably transduced carrying a LTR-driven firefly luciferase as well as a LTR-driven β-galactosidase cassette. Challenging these indicator cells with HIV-1 and HIV-2 isolates results in the induction of luciferase and β-galactosidase allowing easy detection of infection and titration. In the absence of an antibody to measure endogenous or low level TRIM5α expression, a quantitative light-cycler RT-PCR protocol specific for the SPRY-domain was established. Total RNA (2 μg) were used to generate cDNA (superscript II, Invitrogen) using an oligo-dT primer. An aliquot of this cDNA was used as target for the SPRY-specific PCR (primers SP(+): 5'-CCTTTCATTGTGCCCCT-3'; SP(-): 5'-GCACAGAG TCATGGGAC-3') as well as for the β-actin-specific PCR (primers: actin(+): 5'-GGGTCAGAAGGATTCCTATG-3'; actin(-): 5'-GGTCTCAAACATGATCTGGG-3') in order to normalize the cDNA input. The detection limit for both PCR amplifications in the presence of SYBR-green was determined using serial dilutions of plasmids containing the target sequences and revealed a threshold of 10^3 ^molecules per reaction. Using this established qPCR protocol a 2 fold over expression of TRIM5α mRNA in the newly selected TZM-huTRIM5α cells (10384 ± 1032 mRNA molecules versus 5102 ± 531 mRNA molecules in TZM-LNCX2 cells, normalized for β-actin cDNA) was determined. Next, the new indicator cells were challenged with VSV-G pseudotyped B-MLV particles, known to be insensitive to TRIM5α-mediated restriction. Both cell lines were equally well infected using B-MLV particles (550 ng RT per infection as determined using an RT-ELISA, Innovagen, Sweden) transducing a GFP-reporter cassette (51.2% GFP-positive TZM-LNCX2 cells and 50.0% GFP-positive TZM-huTRIM5α, respectively) showing that both cell lines support efficient retroviral infection. The selected cells expressed similar levels of CD4, CXCR4 and CCR5 on the cell surface and maintained a functional tat-inducible firefly luciferase and β-galactosidase reporter cassette like the parental TZM-bl cell line (data not shown), thus are suitable indicator cells to study the influence of human TRIM5α over expression on HIV envelope mediated infection.

**Figure 1:**
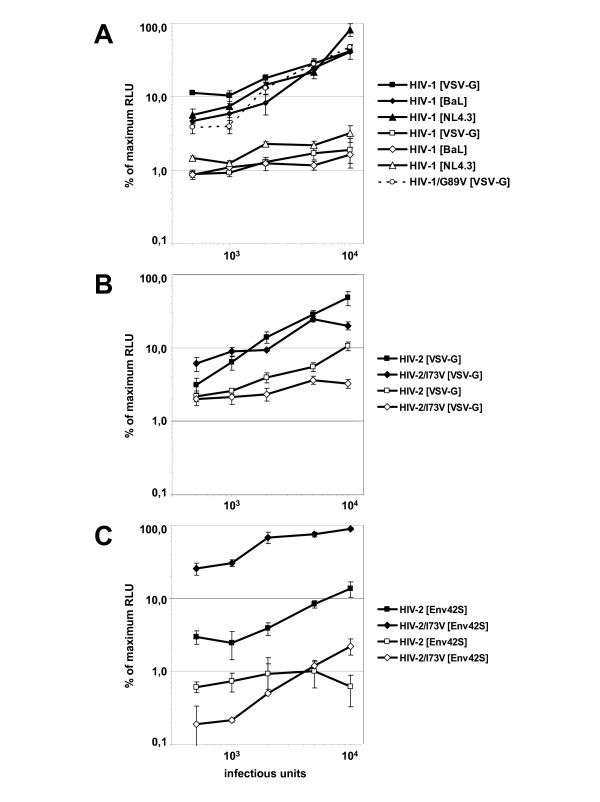
(A) VSV-G envelope and HIV-1 envelope protein pseudotyped viruses are equally restricted by human TRIM5α. Titration of HIV-1[VSV-G], HIV-1[NL4.3] and HIV-1[BaL] viruses onto TZM-LNCX2 cells (closed symbols) and TZM cells expressing human TRIM5α(open symbols) result in 15.3 fold, 12.6 fold and 12.7 fold restriction to infection. (B) VSV-G pseudotyped Lv2-sensitive and Lv2-insensitive HIV-2 viruses are restricted by human TRIM5α. HIV-2[VSV-G] and HIV-2/I73V[VSV-G] viruses were used to infect TZM-LNCX2 cells (closed symbols) and TZM-huTRIM5α cells (open symbols). Human TRIM5α restricted VSV-G mediated HIV-2 infection 3.6 fold and 4.8 fold, respectively. (C) HIV-2 envelope pseudotyped HIV-2 particles reveal entry route dependent Lv2-mediated restriction. HIV-2[Env42S] and HIV-2/I73V[Env42] viruses were used to infect TZM-LNCX2 cells (closed symbols) and TZM-huTRIM5αcells (open symbols). The capsid mutation at position 73 (I73V) confers escape from Lv2-mediated restriction on TZM-LNCX2 cells (9.3 fold increase in infection), whereas the over expression of human TRIM5α in TZM-huTRIM5α cells results in a maximal restriction for both virus variants. Representative results from three independent experiments done in triplicate are shown. All virus preparations were titrated on the parental cell line TZM-bl. Error bars indicate the standard deviations of the data.

First, infection experiments were performed using VSV-G pseudotyped, HIV-1_NL4.3 _envelope and HIV-1_BaL _envelope pseudotyped HIV-1 particles encoding for wild-type capsid using increasing infectious units. TZM-bl cells transduced with the empty vector LNCX2 and G418 selected were used as reference (TZM-LNCX2). The induction of β-galactosidase due to infection of TZM-bl cells (5 × 10^3 ^cells per well) was determined using a luminometer at day 2 post challenge through cell lysis and addition of specific substrates (beta-glo Assay, Promega, Germany). The maximal detectable β-galactosidase activity after challenge of TZM-LNCX2 cells was set to 100% for the different pseudotyped particles (HIV-1[VSV-G], HIV-1[NL4.3], HIV-1[BaL]). As figure 1A shows, the over expression of wild-type human TRIM5α in TZM cells results in substantial restriction to infection for all three viruses to a similar extend. HIV-1[VSV-G] infection was 15.3 fold restricted, whereas the HIV-1 envelope pseudotyped particles showed a 12.6 fold and 12.7 fold restriction for HIV-1[NL4.3] and HIV-1[BaL] respectively. This strong restriction was unexpected, since only a 2 fold over expression of TRIM5α mRNA was detected and previous studies reported only a 2–3 fold restriction of HIV-1 by human TRIM5α [3-8]. However, these studies used cells over expressing HA-tagged TRIM5α, which in the case of rhesus TRIM5α has been described to be less efficient in restricting SIV_mac _infection [7]. Whether the HA-tagged TRIM5α is less stable or less active than wild type TRIM5α or other factors differ between TZM-bl cells and HeLa cells influencing retroviral restriction efficiency needs to be further elucidated. However, the results obtained clearly indicate that human TRIM5α is capable to restrict HIV-1 infection quite substantially but that the restriction due to TRIM5α is entry route independent (VSV-G versus HIV-1 envelope) and HIV coreceptor independent (X4-tropic versus R5 tropic).

Next, the restriction of HIV-2 infection due to human TRIM5α expression in TZM cells was analyzed. Like for the pseudotyped HIV-1 particles, HIV-2 reporter viruses encoding for renilla luciferase (similar to the HIV-1 reporter viruses used before) were generated through transfection of 293T cells with the proviral ROD/A-ΔenvRen plasmid and the expression plasmid for either VSV-G or Env42S envelope protein (MP11-VSV-G and MP11-Env42S, respectively) [22]. MP11-Env42S encodes for the envelope protein of the TCLA isolate HIV-2_CBL23_. In addition, a Lv2-insensitive HIV-2 variant was constructed. The proviral ROD/A-ΔenvRen plasmid (encoding isoleucine at position 73 of the capsid protein, shown to cause a Lv2-sensitive phenotype in the context of the molecular clone HIV-2_MCR_) was mutagenized to exchange isoleucine at position 73 to valine resulting in a Lv2-insensitive HIV2_ROD _variant (HIV-2/I73V) similar to HIV-2_MCN _[11]. The resulting proviral plasmid (ROD/A/I73V-ΔenvRen) was used to generate VSV-G and Env42S envelope pseudotyped particles. Using increasing infectious doses to challenge TZM-huTRIM5α cells a 3.4 and 4.8 fold restriction of VSV-G pseudotyped HIV-2 and HIV-2/I73V particles could be determined (fig. 1B). This result is in agreement with earlier studies using CRFK cells expressing human TRIM5α after challenge with VSV-G pseudotyped HIV-2_ROD _[9] but shows in addition that the Lv2-insensitive HIV-2/I73V remains restricted by human TRIM5α.

The challenge experiments with HIV-2 envelope protein Env42S pseudotyped HIV-2 particles (HIV-2[Env42S] and HIV-2/I73V[Env42S]) however confirmed again our previous observation that the Lv2-mediated restriction is entry route dependent [10,11,22]. As figure 1C shows, the over expression of human TRIM5α in TZM cells results in a 2.5 times stronger restriction to infection for Env42S-pseudotyped HIV-2 particles (8.6 fold restriction) compared to VSV-G pseudotyped HIV-2 particles (3.4 fold restriction). For HIV-2/I73V[Env42S] a 9.3 fold increase in infection of TZM-LNCX2 cells compared to HIV-2[Env42] was observed, indicating the escape from Lv2-mediated restriction due the single amino acid change in the capsid protein. Compared to the control cells TZM-LNCX2 the over expression of human TRIM5α resulted in a 80.6 fold restriction to infection. However, since the restriction of HIV-2/I73V[Env42S] on TZM-huTRIM5α cells was not changed compared to HIV-2[Env42S] one can conclude again that the Lv2-insensitive HIV-2/I73V remains restricted by human TRIM5α. Furthermore, the only 2 fold increase of human TRIM5α mRNA in TZM-huTRIM5α cells is sufficient to confer a maximal restriction, even for the Lv2-insensitive HIV-2/I73V variant.

**Figure 2:**
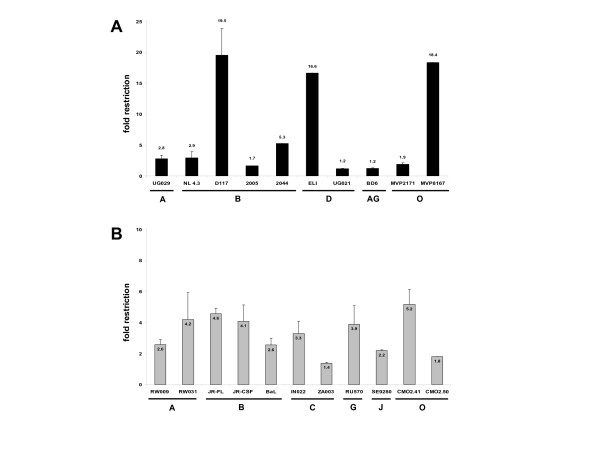
Human TRIM5α mediated restriction varies between 1.2 and 19.5 fold independent of subtype or coreceptor usage. Different primary isolates of HIV-1 subtype A, B, C, D, G, J, CFR_AG and HIV-1 group O (2 × 10^3 ^infectious units per well) were used to infect TZM-huTRIM5α cells and the relative restriction to infection compared to TZM-LNCX2 cells was calculated. CXCR4 tropic (A) and CCR5-tropic (B) virus isolates and molecular cloned viruses were used. Three independent experiments were done in triplicate. Error bars indicate the standard deviations of the data.

In order to analyse the human TRIM5α mediated restriction of primary isolates and molecular clones of different HIV-1 subtypes (A to D, G, J, CRF_AG and HIV-1 group O) (obtained through the NIH AIDS Research and Reference Reagent Program or described in further detail in [23-25]) the new indicator cells TZM-huTRIM5α and the control cells TZM-LNCX2 were challenged with 2 × 10^3 ^infectious units, as titrated on parental TZM-bl cells (equals a MOI of 0.2), and again the induction of β-galactosidase two days post infection was determined. As figure 2 shows, some HIV-1 isolates tested were only marginally restricted (1.2 to 1.4 fold for UG021, BD6 and ZA003) whereas the vast majority of isolates was restricted between 2.2 and 5.2 fold. Three exceptional strong restricted isolates could be identified, namely D117 (subtype B), ELI (subtype D) and MVP8167 (group O), being restricted between 16.6 and 19.5 fold compared to the control cells TZM-LNCX2. These three primary isolates are CXCR4-tropic variants. However, the mean restriction to infection for the remaining 18 isolates tested was 3.0 ± 1.3 fold, indicating that there are no significant coreceptor-specific differences between the X4-tropic (mean 2.5 ± 1.5 fold restriction for 7 isolates) and R5-tropic (mean 3.2 ± 1.2 fold restriction for 11 isolates) variants studied. In comparison to the experiments performed with pseudotyped particles, a weaker restriction to infection with HIV-1_NL4.3 _versus HIV-1[NL4.3] was observed. NL4.3 envelope pseudotyped particles derived from 293T transfections resulted in a higher ratio of infectious units per ng RT/ml as compared to HIV-1_NL4.3 _virus stocks obtained from PBMC cultures. Therefore, PBMC derived virus stocks might contain a larger proportion of virus-like particles able to abrogate TRIM5α, resulting in a weaker restriction to infection, which could explain the observed difference in restriction efficiency. However, the quantity of virus-like particles per virus preparation for the other virus stocks used is not known and difficult to address. As for the three outliers in this study it is tempting to speculate that they might not only be restricted by TRIM5α but also by Lv2 or yet another unknown restriction factor, as we could show in this study that both TRIM5α and Lv2 restriction factors can act on incoming HIV-2 capsids. However, further studies are needed together with the identification of the restriction factor Lv2.

Taken together our results show that even a moderate over expression of wild-type human TRIM5α in human cells (2 fold as determined by quantitative RT-PCR) confers substantial restriction to infection for HIV-1 (12.7 fold restriction for pseudotyped HIV-1 particles) but only a weaker restriction to infection for HIV-2 (between 3.4 and 4.8 fold restriction for pseudotyped HIV-2 particles). This overall stronger restriction to infection described here compared to previous reports [3-8] could be explained by non-tagged human TRIM5α being more stable than the HA-tagged variant most often used in those studies. There is also the possibility that the HA-tag on TRIM5α causes a reduction in the activity as a restriction factor, as has been described for the rhesus TRIM5α variant [7]. In addition, other unidentified factors that differ between Hela-cells and TZM-bl cells could account for the observed stronger restriction and need to be further characterized. The challenge experiments using Lv2-sensitive and Lv2-insensitive HIV-2 variants revealed that both Lv2 and human TRIM5α act together on the incoming HIV-2 capsid and that the 2 fold over expression of TRIM5α in TZM-TRIM5α cells is sufficient to confer a maximal restriction to infection. Since Lv2 has not been identified yet, the endogenous level of Lv2 can not be determined. However, the endogenous level of Lv2 in TZM-LNCX2 cells is sufficient confer a 8.6 fold restriction to infection, indicating that Lv2 is a potent restriction factor. It has been described that certain HIV-1 variants are also restricted by Lv2 [12]. Whether the three HIV-1 isolates D117, ELI and MVP8167 identified as being more efficiently restricted in TZM-huTRIM5α cells are in addition susceptible to Lv2-mediated restriction or restricted by yet another unidentified factor needs to be further elucidated. There is no obvious sequence similarity between HIV-1 and HIV-2 capsid around amino acid position 73, where Lv2 susceptibility has been mapped to. However, differences in viral uptake or differences in activation of target cells due to envelope binding, leading to more or less active restriction factors, could also explain the observed strong restriction efficiency for these three primary HIV-1 isolates and merit further investigations.

## Abbreviations

HIV-1, HIV-2, human immunodeficiency virus type 1 and type 2; TRIM, tripartite motif protein; HA-tag, epitope mapping to an internal region of influenza hemaglutinin protein; VSV-G, vesicular stomatitis virus glycoprotein; Lv2, lentivirus restriction factor 2; TCLA, tissue culture lab adapted

## Competing interests

The author(s) declare that they have no competing interests.

## Authors' contributions

PK and MTD conceived the experiments and wrote the manuscript. PK, IH and MTD performed the laboratory work. All authors read and approved the final manuscript.
